# Synovial Sarcoma Mimicking Haemophilic Pseudotumour

**DOI:** 10.1155/SRCM/2006/27212

**Published:** 2006

**Authors:** Haroon A. Mann, Andrew Hilton, Nicholas J. Goddard, Michael A. Smith, Brian Holloway, Christine A. Lee

**Affiliations:** ^1^Department of Trauma & Orthopaedics, Royal Free Hampstead NHS Trust, London NW2 5QX, UK; ^2^Department of Trauma & Orthopaedics, St Thomas' Hospital, London SE1 7EH, UK; ^3^Department of Radiology, Royal Free Hampstead NHS Trust, London NW2 5QX, UK; ^4^Haemophilia and Haemostasis Unit, Katherine Dormandy Centre, Royal Free Hampstead NHS Trust, London NW2 5QX, UK

## Abstract

This is a case of a 36-year-old gentleman with haemophilia A who was presented with an acute atraumatic soft tissue swelling in the right thigh. Open biopsy was performed with the resultant diagnosis of a synovial cell sarcoma. Although the clinical findings were nonspecific they could easily have been found in a bleeding haemophilic pseudotumour. The findings reported on MRI scan initially were highly consistent with those present in patients with mild haemophilia. An important part of orthopaedic management in haemophilia is concerned with intraarticular and intramuscular bleeding. Haematomas are common and sarcomas are rare. However the absence of trauma should alert the clinician to the possibility that the abnormality may represent haemorrhage into a tumour and not just haematoma, even in a haemophilic patient.

## INTRODUCTION

Soft tissue sarcomas account for approximately 5−10% of all mesenchymal malignancies. Synovial cell sarcomas are the commonest of the soft tissue sarcoma especially in people aged between fifteen and forty years. Overall there are two hundred new cases in the UK and eight hundred in the USA presenting to clinicians annually.

We present an unusual case of a synovial cell sarcoma in a patient with mild haemophilia A.

## CASE REPORT

A 36-year-old Sudanese gentleman with mild haemophilia A (F VIII : C level 38 iu/dl/normal range > 50 iu/dl) presented with a five-week history of an atraumatic cystic swelling in the anterior aspect of the right thigh. Clinically this cystic lesion was typical of a haemophilic pseudotumour.

On examination there was an 8 × 10 cm mass in the anterior compartment of the right thigh which was tender to palpation.

**Figure 1:**
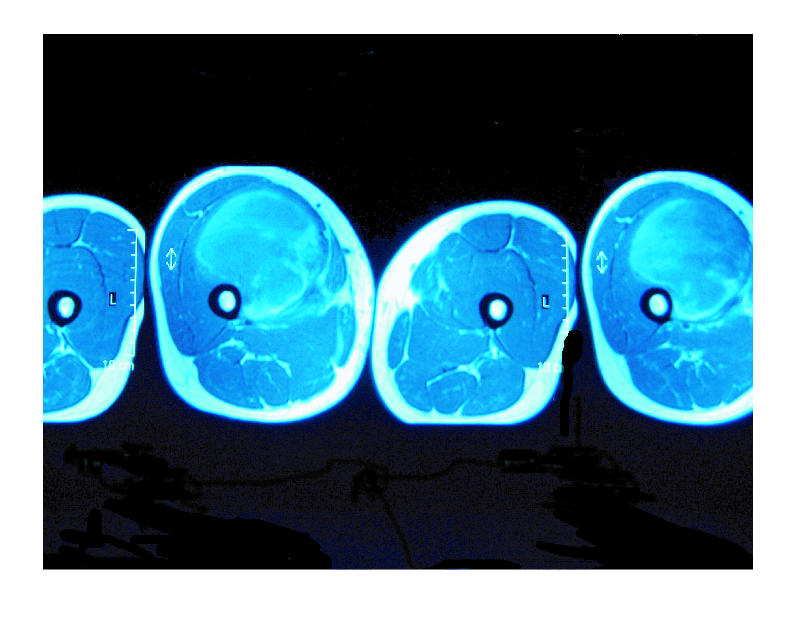
T1-weighted axial spin-echo pulse sequence. Huge mass seen in the right anterior thigh. Peripheral high signal is in keeping with methaemoglobin.

**Figure 2:**
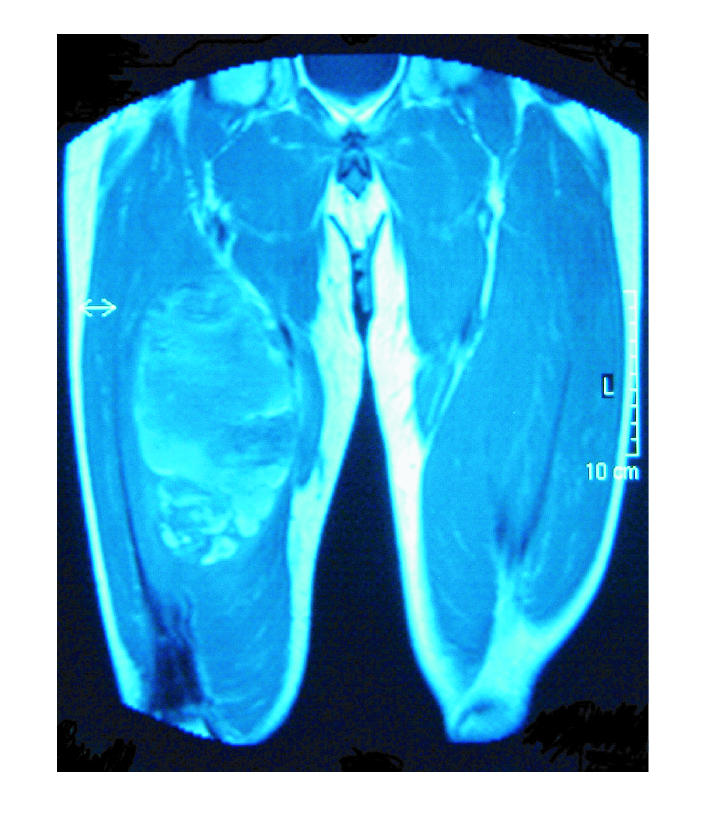
T1-weighted coronal spin-echo pulse sequence. Peripheral high signal is seen within the entire lesion.

**Figure 3:**
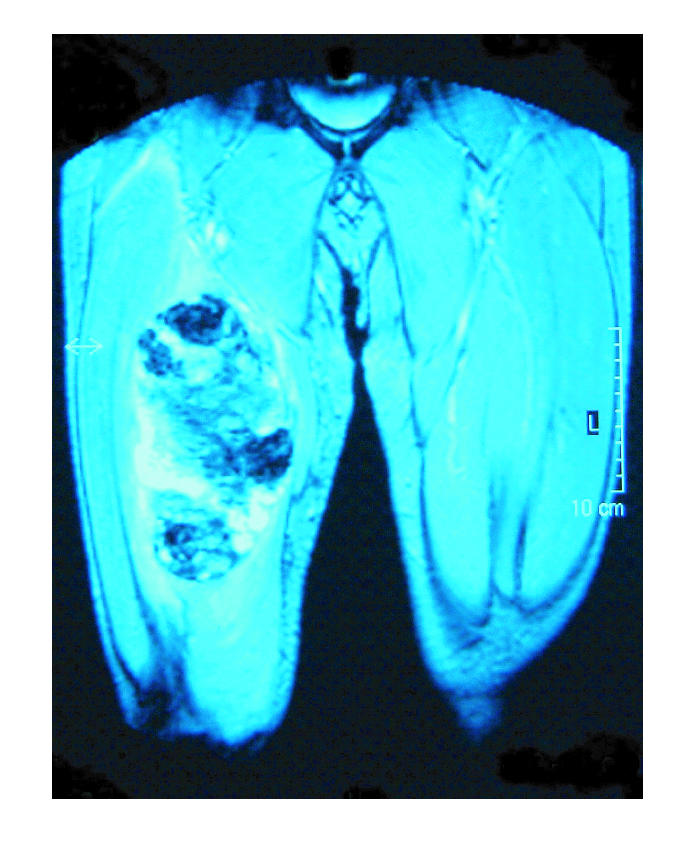
T2-weighted gradient-echo pulse sequence. Marked low signal is seen centrally secondary to magnetic susceptibility artefact from haemosiderin.

Ultrasound scan examination revealed a large heterogeneous mass of mixed echotexture with a central area of liquefaction consistent with an intramuscular haematoma. A subsequent angiogram showed no evidence of vessels supplying the lesion or the presence of arteriovenous malformations. MRI scanning confirmed an 18 × 10 × 11 cm loculated cystic lesion in the anterior compartment of the thigh involving the vastus intermedius and vastus medialis muscles. T1 weighting (T1W) demonstrated a lesion with peripheral high signal (Figure 1). Gradient-echo T2-weighted images revealed marked signal drop out centrally due to magnetic susceptibility artefact from altered blood products, usually haemosiderin (Figure 2). The mass was shown to extend posteriorly as far as the medial cortex of the femur and medially to the femoral canal (Figure 3). No extension into bone was seen. It was concluded that appearances were consistent with those of an organising haematoma. Given the past medical history, a provisional diagnosis of haemophilic pseudotumour was made.

Standard conservative treatment was commenced with a continuous infusion of factor VIII, analgesia, and bed rest. Despite this, the pain and swelling increased over the next five days and it was decided to evacuate the haematoma. The haematoma was excised and approximately one litre of freshly clotted and organised blood was drained. Haemostasis was achieved and the entire cavity packed. However, over the following seven days the swelling in the right thigh recurred. A further MRI scan was performed and the findings were once more consistent with further haematoma formation. Approximately 1 litre of clotted blood was drained under ultrasound guidance.

Six weeks after the initial presentation the swelling recurred with an overall increase in size. It was decided to perform an open exploration, and a biopsy was taken of the cyst wall. Subsequent histology revealed poorly differentiated synovial sarcoma. Staging of the tumour was performed with a CT of the thorax which demonstrated pulmonary metastatic disease.

The tumour was found to be unresectable and a complete disarticulation of the hip was subsequently performed. The patient went on to a course of combination chemotherapy.

## DISCUSSION

Synovial cell sarcoma is a relatively frequent soft tissue sarcoma. It is the fifth most common soft tissue malignancy and is one of the most common soft tissue sarcomas in adolescents and young adults, presenting in some 13% of soft tissue malignancy. Synovial cell sarcomas are a misnomer in that they do not arise from synovium. They are so named due to their resemblance to synovium on light microscopy.

The presentation is most commonly between 15 and 35 years but has been reported in children and even in neonates [1, 2]. Patients may present with symptoms of pain or a palpable mass slowly increasing in size. The extremities have a preponderance to synovial sarcomas with up to 90% occurring at these sites. The lower limb is a frequent site, with 60−70% of tumours occurring in the thigh or calf. The popliteal fossa is also frequently involved [3, 4]. Less common sites include the head and neck, thorax, pelvis, and the paravertabral regions.

Radiographs may be normal but up to 50% of cases show the presence of a nonspecific mass [5], 25% of tumours have areas of calcification, typically in the periphery of the lesion. Up to 20% of tumours may involve adjacent bone, evidenced as periosteal reaction, extrinsic erosion or aggressive osseous invasion. On MRI and CT scan, synovial sarcoma is usually seen as a heterogeneous mass. CT frequently shows necrosis and haemorrhagic areas of lower attenuation with other areas of soft tissue attenuation similar to that of muscle.

Haemophilic pseudotumours are a rare but serious condition in patients with haemophilia. They present as progressive cystic swellings encapsulating a haematoma and commonly involving muscles adjacent to bones to muscles in the proximal skeleton [6]. They are classified according to their location as subcutaneous, intramuscular, interfascial, subperiosteal, and intraosseous. Their pathology is described in accordance to haematomas in various stages of resolution and occasionally by new haemorrhage within areas of fibrous organisation.

Features characteristic to both haemophilic pseudotumors and soft tissue sarcomas may be nonspecific and therefore pose a potential diagnostic dilemma. The MRI signal characteristics of soft tissue haemorrhage depend on the age of the haemorrhage. In the acute stage (1–6 days) an intermediate signal intensity on T1W images and low signal intensity on T2W images is seen. After the first week, haemoglobin is oxidised to methaemoglobin leading to high intensity signal on T1W. T2W images may be of either low (due to intracellular methaemoglobin) or high (due to extracellular methaemoglobin) signal intensity. Gradient-echo pulse sequences, often employed in magnetic imaging are prone to magnetic susceptibility artefact. This occurs with materials which have paramagnetic properties, the commonest of which is chronic blood products.

Soft tissue sarcomas mimicking haematomas have been described previously [7–9]. To the best of our knowledge soft tissue sarcomas mimicking as pseudotumours have only been reported in the literature on two previous occasions [10, 11]. Diagnosis without open biopsy is extremely difficult since techniques such as percutaneous aspiration have a low yield of tumour cells from the haematoma [9].

The treatment of synovial sarcoma is often amputation of the limb, although wide enblock resection and limb salvage may also be performed. Up to 25% of patients present with metatstatic disease at diagnosis and despite aggressive therapy metatstatic lesions occur in up to 80% of patients [12]. 60−90% of metastases occur in the lungs, 5−10% in lymph nodes, and 8−10% in bones. Local recurrence is common within two years of initial presentation. Five-year survival can be anything from 27−55%. [4, 13]. The most important factors determining prognosis are early diagnosis and small (< 5 cm in diameter) tumour size [4]. Other favourable prognostic features include extensive calcification, younger age, and lesions located in the extremities.

## CONCLUSION

This is a case of a 36-year-old gentleman with haemophilia A who presented with an acute atraumatic soft tissue swelling in the right thigh (Figures 1, 2, 3). Open biopsy was performed with the resultant diagnosis a synovial cell sarcoma. Although the clinical findings were nonspecific they could easily have been found in a bleeding haemophilic pseudotumour. An important part of orthopaedic management in haemophilia is concerned with intraarticular and intramuscular bleeding. Haematomas are common and sarcomas are rare. However the absence of trauma should alert the clinician to the possibility that the abnormality may represent haemorrhage into a tumour and not just haematoma, even in a haemophiliac.
